# Supplementary material for: Prefrontal gamma oscillations and fear extinction learning require early postnatal interneuron-oligodendroglia communication
Source: Nat Commun. 2025 Dec 18;16:11225. doi: 10.1038/s41467-025-66309-3 (PMC12715222; doi:10.1038/s41467-025-66309-3)
Supplement: Supplementary file 1 — Supplementary Information [file 41467_2025_66309_MOESM1_ESM.pdf]

Suplementary Information

Plaisier et al.

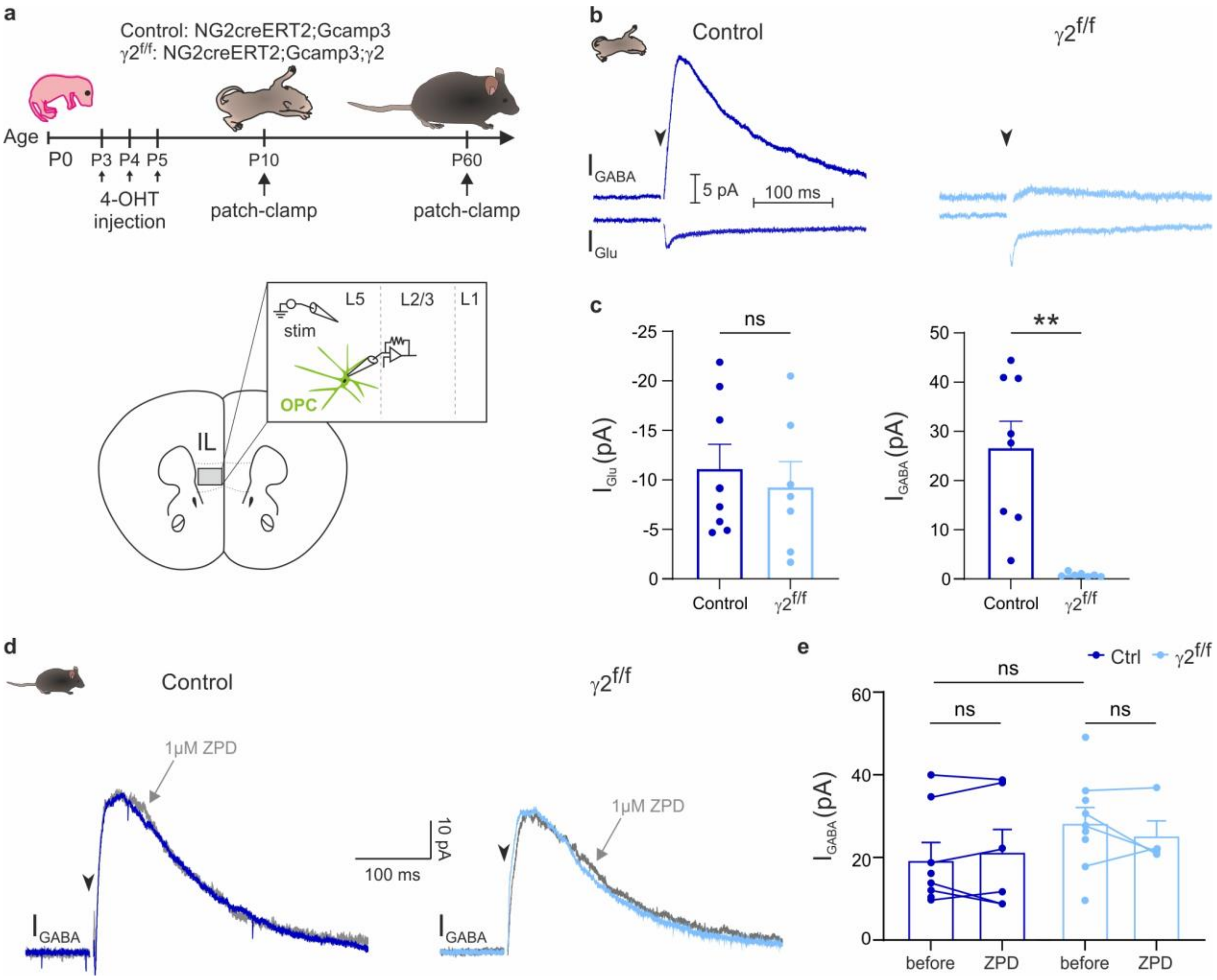

Supplementary Figure 1. Elimination of γ2-GABA$_A$ receptor-mediated postsynaptic currents in OPCs of the mPFC in pups

a. Experimental protocol. Both control and γ2$^{f/f}$ mice were injected with 4-OHT from P3 to P5 followed by patch-clamp recordings of OPCs in acute mPFC slices at around P10 (P9-P13), coinciding with the peak of cortical GABAergic synaptic connectivity[15], or at around P60 (P59-P78). **b.** Representative evoked glutamatergic (I_{Glu}) and GABAergic (I_{GABA}) postsynaptic currents in OPCs of control (left) and γ2$^{f/f}$ (right) mice in response to extracellular stimulation

of layer 5 of the IL region of the mPFC at P9-P13. Note the near-complete abolishment of I_{GABA} in γ2$^{f/f}$ mice. Stimulation artifacts blanked, stimulation time indicated (black arrowheads). **c.** Dot plots showing the amplitudes of I_{Glu} (left) and I_{GABA} (right) in control (dark blue; n=8 cells from N=3 mice) and γ2$^{f/f}$ (light blue; n=7 cells from N=3) mice. p= 0.6107 for I_{Glu}; p= 0.0030 for I_{GABA}; two-tailed Student's *t* test for excitation and two-tailed Welch's t test for inhibition. Data are represented as mean±s.e.m. **p<0.01, ns: not significant. **d.** Representative evoked GABAergic (I_{GABA}) postsynaptic currents in OPCs of control (left) and γ2$^{f/f}$ (right) mice in response to extracellular stimulation of layer 5 of the IL region of the mPFC at P59-P78. Recordings were obtained in normal conditions (blue traces) and in the presence of 1 μM zolpidem (ZPD; gray traces). Stimulation artifacts blanked, stimulation time indicated (black arrowheads). **e**. Dot plots showing the amplitudes of I_{GABA} before and after 4 min of bath application of ZPD in control (dark blue: n=8 cells in total and n=6 in ZPD from N=3 mice) and γ2$^{f/f}$ mice (light blue: n=7 cells in total and n=4 in ZPD from N=3 mice) mice. Note that, unlike during development, evoked I_{GABA} were similar in control and γ2$^{f/f}$ mice at P59-P78 and were not significantly potentiated by zolpidem (1 μM), indicating the absence of functional γ2-containing GABA-A receptors in adult OPCs. p= 0.1996 for comparison of I_{GABA} in normal conditions, p>0.9999 for comparison of ZPD potentiation in control mice; p= 0.6250 for comparison of ZPD potentiation in γ2$^{f/f}$ mice; two-tailed Mann-Whitney U test to compare I_{GABA} in control and γ2$^{f/f}$ mice; Wilcoxon test for ZPD. Data are represented as mean±s.e.m. ns: not significant. 4-OHT: 4-hydroxytamoxifen. Source data are provided as a Source Data file.

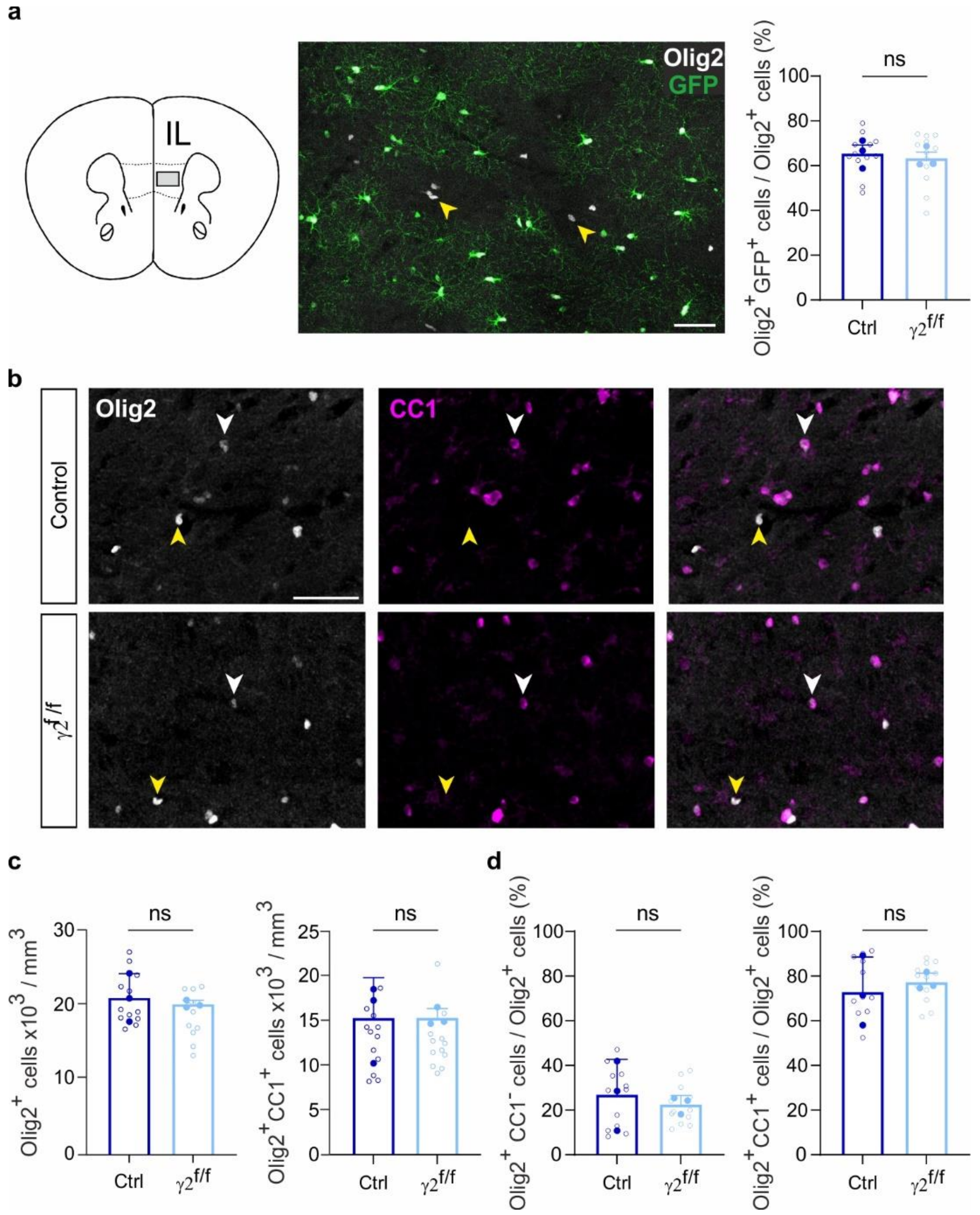

Supplementary Figure 2. Recombination efficiency and no changes in the density and proportions of Olig2$^+$/CC1$^-$ OPCs and Olig2$^+$/CC1$^+$ OL of the mPFC in γ2$^{f/f}$ mice

a. Confocal image (middle) of Olig2$^+$ (white) and GFP$^+$ (green) cells in IL region of the adult mPFC (left). Note that most Olig2$^+$ cells are positive for GFP. Some Olig2$^+$/GFP$^-$ cells are

indicated (yellow arrowheads). Scale bar: 50 µm. Dot plots (right) of the recombination efficiency for control (dark blue) and $\gamma2^{f/f}$ (light blue) mice. Control mice: n= 12 technical replicates from N= 3 mice (biological replicates) and $\gamma2^{f/f}$ mice: n= 12 technical replicates from N= 3 mice (biological replicates); p= 0.598 for (**a**); two-tailed linear mixed models with likelihood ratio tests applied to account for repeated measures (open dots) within each animal (closed dots). Data are represented as mean±s.e.m. ns: not significant**. b.** Representative confocal images of $Olig2^+$ (white) and $CC1^+$ (magenta) cells in IL region of the mPFC (inset) in four-month control (top) and $\gamma2^{f/f}$ (bottom) mice. Arrowheads indicate $Olig2^+/CC1^+$ OL cells (white) and $Olig2^+/CC1^-$ OPCs (yellow). Scale bar: 50 µm. **c.** Quantification of $Olig2^+$ and $Olig2^+/CC1^+$ cell densities in control (dark blue) and $\gamma2^{f/f}$ mice (light blue). Control mice: n= 12 technical replicates from N= 3 mice (biological replicates) and $\gamma2^{f/f}$ mice: n= 12 technical replicates from N= 3 mice (biological replicates). p= 0.615 for $Olig2^+$ and p= 0.915 for $Olig2^+/CC1^+$ quantifications; two-tailed linear mixed models with likelihood ratio tests applied to account for repeated measures (open dots) within each animal (closed dots). Data are represented as mean±s.e.m. ns: not significant. **d.** Ratio of $Olig2^+/CC1^-$ OPCs and $Olig2^+/CC1^+$ OL cell densities compared to the total number of $Olig2^+$ cells in control (dark blue) and $\gamma2^{f/f}$ mice (light blue). Control mice: n= 12 technical replicates from N= 3 mice (biological replicates) and $\gamma2^{f/f}$ mice: n= 12 technical replicates from N= 3 mice (biological replicates). p= 0.624 for OPC and p= 0.624 for OL cells quantifications; two-tailed linear mixed models with likelihood ratio tests applied to account for repeated measures (open dots) within each animal (closed dots). Data are represented as mean±s.e.m. ns: not significant. Source data are provided as a Source Data file.

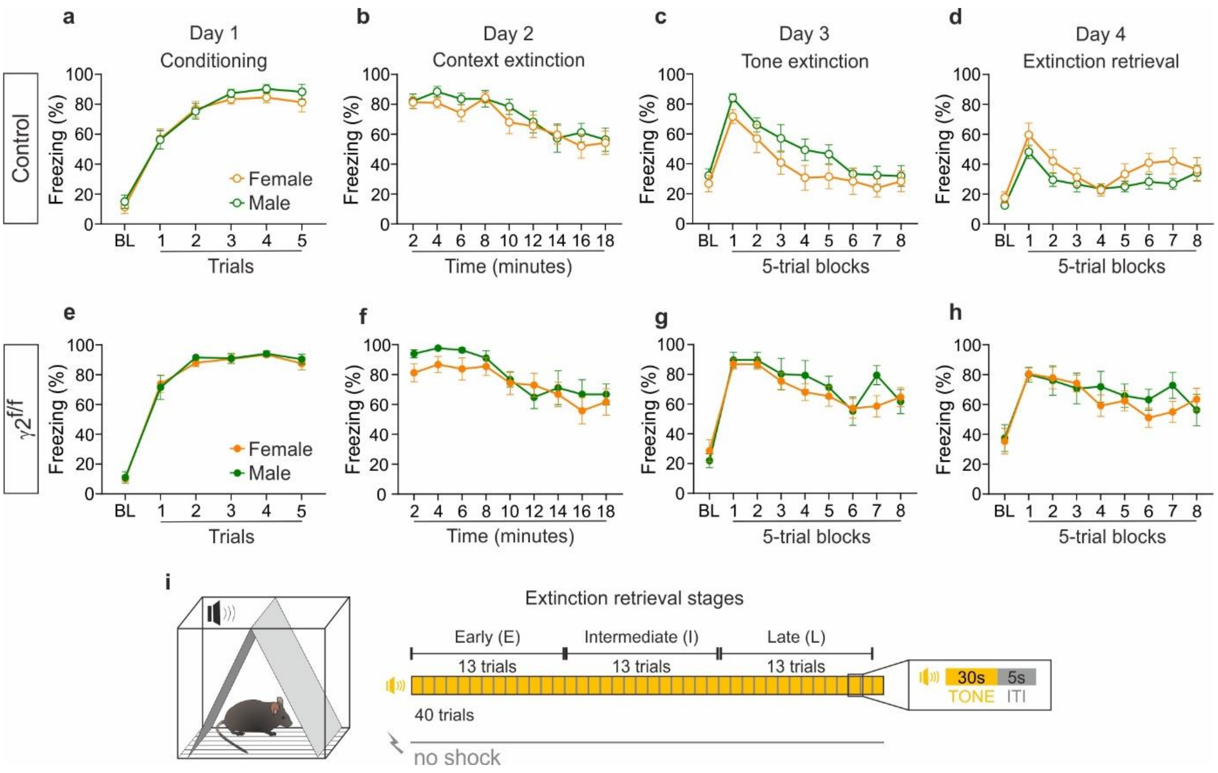

Supplementary Figure 3. No sex-dependent behavioral differences observed in control and γ2$^{f/f}$ mice

a-h. Mice were subjected to a four-day fear conditioning protocol consisting of four sessions (see Fig. 1 for the protocol). Comparison of freezing behavior between males (green) and females (orange) in control (**a-d**) and γ2$^{f/f}$ (**e-h**) mice during each session. No significant sex-related differences were observed in either group (control mice: N=9 males and N=9 males females; γ2$^{f/f}$ mice: N=8 males and N=15 females). p= 0.9951 / >0.9999 / >0.9999 / 0.9311 / 0.7790 / 0.9555 for BL and trials 1-5 in (**a**); p= >0.9999 / 0.7735 / 0.8212 / >0.9999 / 0.9519 / >0.9999 / >0.9999 / 0.9876 / >0.9999 at 2-18 min in (**b**); p= 0.9968 / 0.3125 / 0.9905 / 0.8630 / 0.6554 / 0.7887 / 0.9998 / 0.9728 / >0.9999 for BL and 5-trial blocks 1-8 in (**c**); p= 0.9310 / 0.9032 / 0.8360 / 0.9993 / >0.9999 / 0.9680 / 0.7230 / 0.7247 / >0.9999 for BL and 5-trial blocks 1-8 in (**d**); p= >0.9999 / >0.9999 / 0.8630 / >0.9999 / >0.9999 / 0.9965 for BL and trials 1-5 in (**e**); p= 0.4722 / 0.4544 / 0.6724 / 0.9971 / >0.9999 / 0.9966 / >0.9999 / 0.9920 / >0.9999 for 2-18 min in (**f**); p= 0.9958 / >0.9999 / >0.9999 / >0.9999 / 0.9753 / 0.9993 / >0.9999 / 0.3224

/ >0.9999 for BL and 5-trial blocks 1-8 in (**g**); p= >0.9999 / >0.9999 / >0.9999 / >0.9999 / 0.9748 / >0.9999 / 0.8924 / 0.7174 / 0.9996 at BL/5-trial blocks 1-8 in (**h**); two-way ANOVA test followed by post-hoc Sidak multiple comparisons test. Data are represented as mean±s.e.m.

i. Schematic of the early, intermediate and late stages (13 tones for each stage) of the tone extinction retrieval session. Source data are provided as a Source Data file.

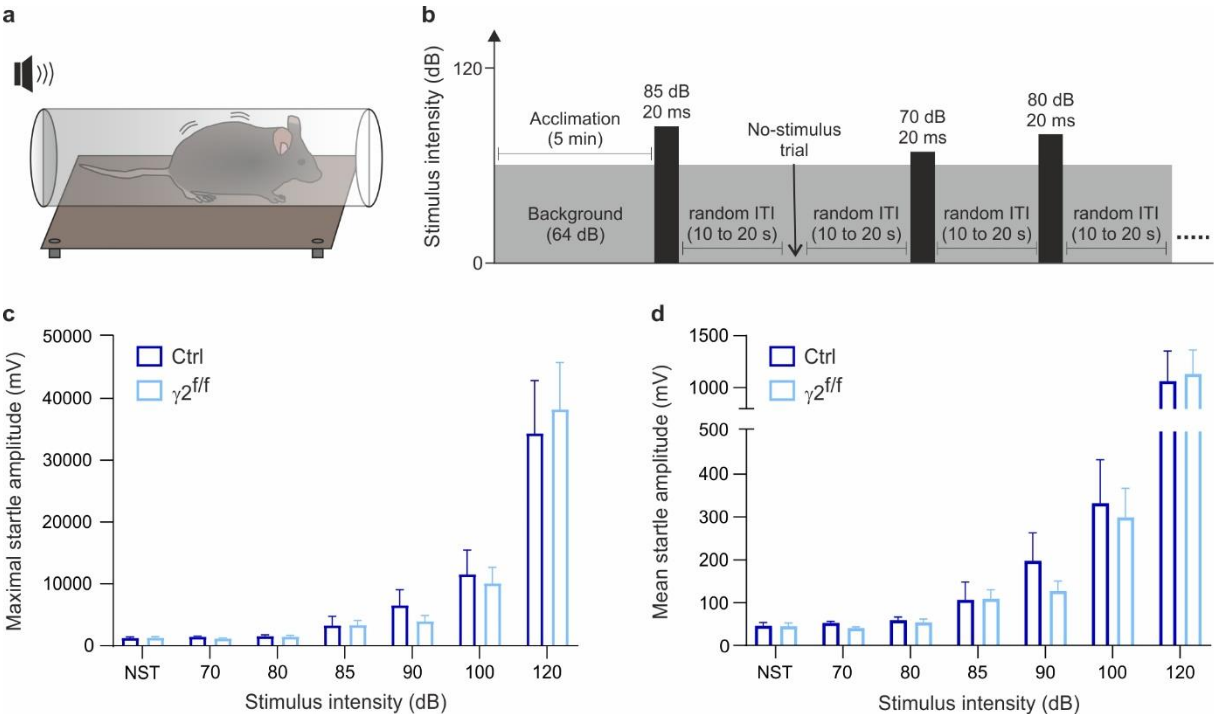

Supplementary Figure 4. No auditory impairments detected in γ2$^{f/f}$ mice

a,b. A mouse in the startle apparatus (**a**) was exposed to tone stimuli of varying intensities (**b**, measured in dB) and lasting 20 ms, with an inter-trial interval of 10 to 20 seconds. Following 5 min of acclimation period under a constant background noise of 64 dB, the stimuli were presented in random order. **c,d.** Startle response to the randomly applied tone stimuli of varying intensities. No significant differences were observed for either the maximal startle amplitude (**c**) or the mean startle amplitude (**d**). N=8 control mice and N=8 γ2$^{f/f}$ mice. p= >0.9999 / >0.9999 / >0.9999 / >0.9999 / 0.9981 / >0.9999 / 0.9785 for NST, 70, 80, 85, 90, 100, 120 dB in (**c**); p= >0.9999 / >0.9999 / > 0.9999 / >0.9999 / 0.9993 / >0.9999 / 0.9992 for NST, 70, 80, 85, 90, 100, 120 dB in (**d**); mixed-effects model followed by a Sidak's multiple comparison test. Data are represented as mean±s.e.m. NST: No-stimulus trial. Source data are provided as a Source Data file.

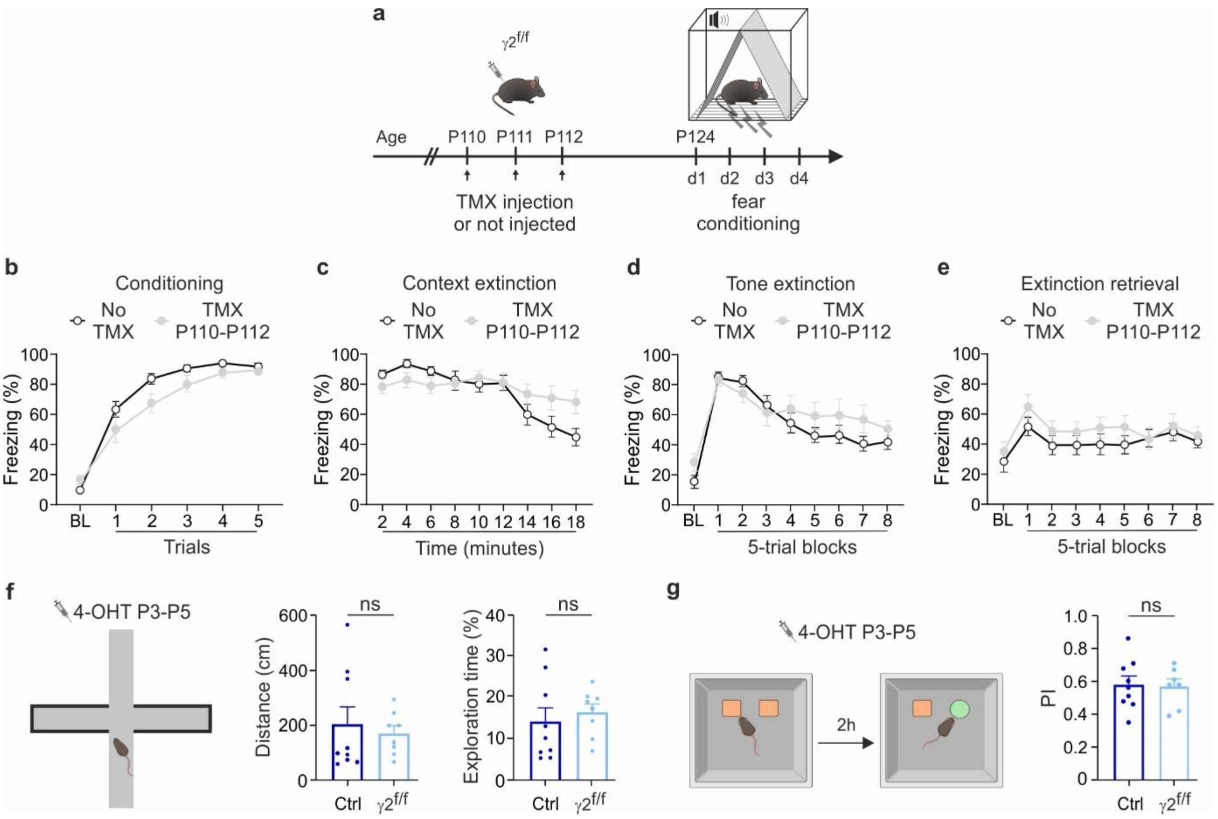

Supplementary Figure 5. Comparable behavioral performance in $\gamma2^{f/f}$ mice following adult recombination or during elevated plus maze and novel object recognition following early postnatal recombination.

a-e. Mice received tamoxifen (TMX) injections for three consecutive days two weeks prior to be subjected to the same fear condition protocol of Fig. 1a. Comparisons of freezing behavior are shown between $\gamma2^{f/f}$ mice that were not injected (black) and those that received injections in adulthood (gray) for each session. No significant differences were observed between the two groups. N=15 and N=12 for not injected and injected in adulthood $\gamma2^{f/f}$ mice, respectively; p= 0.3115 / 0.7434 / 0.2140 / 0.4495 / 0.3692 / 0.9955 for BL and trials 1-5 in (**b**); p= 0.8014 / 0.5073 / 0.7394 / >0.9999 / 0.9991 / >0.9999 / 0.8445 / 0.5385 / 0.1911 for 2-18 min in (**c**); p= 0.5495 / >0.9999 / 0.9432 / 0.9998 / 0.9908 / 0.8998 / 0.9443 / 0.6961 / 0.9849 for BL and 5-trial blocks 1-8 in (**d**); p= 0.9978 / 0.8794 / 0.9716 / 0.9846 / 0.9463 / 0.8884 / >0.9999 / >0.9999 / 0.9997 for BL and 5-trial blocks 1-8 in (**e**); two-way ANOVA test followed by post-hoc Sidak

multiple comparisons test. Data are represented as mean±s.e.m. **f, g.** Following 4-OHT administration (P3–P5), mice were subjected to an elevated plus maze (EPM, **f**) or novel object recognition test (NOR, **g**). No significant differences were observed either for the distance and exploration time (%) during the EPM or the preference index (PI) during the NOR between control and $\gamma 2^{f/f}$ mice. EPM: N=9 for control and N=8 for $\gamma 2^{f/f}$ mice; NOR: N=9 for control and N=7 for $\gamma 2^{f/f}$ mice. p= 0.6058 for distance (**f;** two-tailed Mann-Whitney U test), p= 0.5788 for exploration time (**f**), p= 0.8533 for PI (**g**), two-tailed Student's t test. All data are represented as mean±s.e.m. ns: not significant. 4-OHT: 4-hydroxytamoxifen. Source data are provided as a Source Data file.

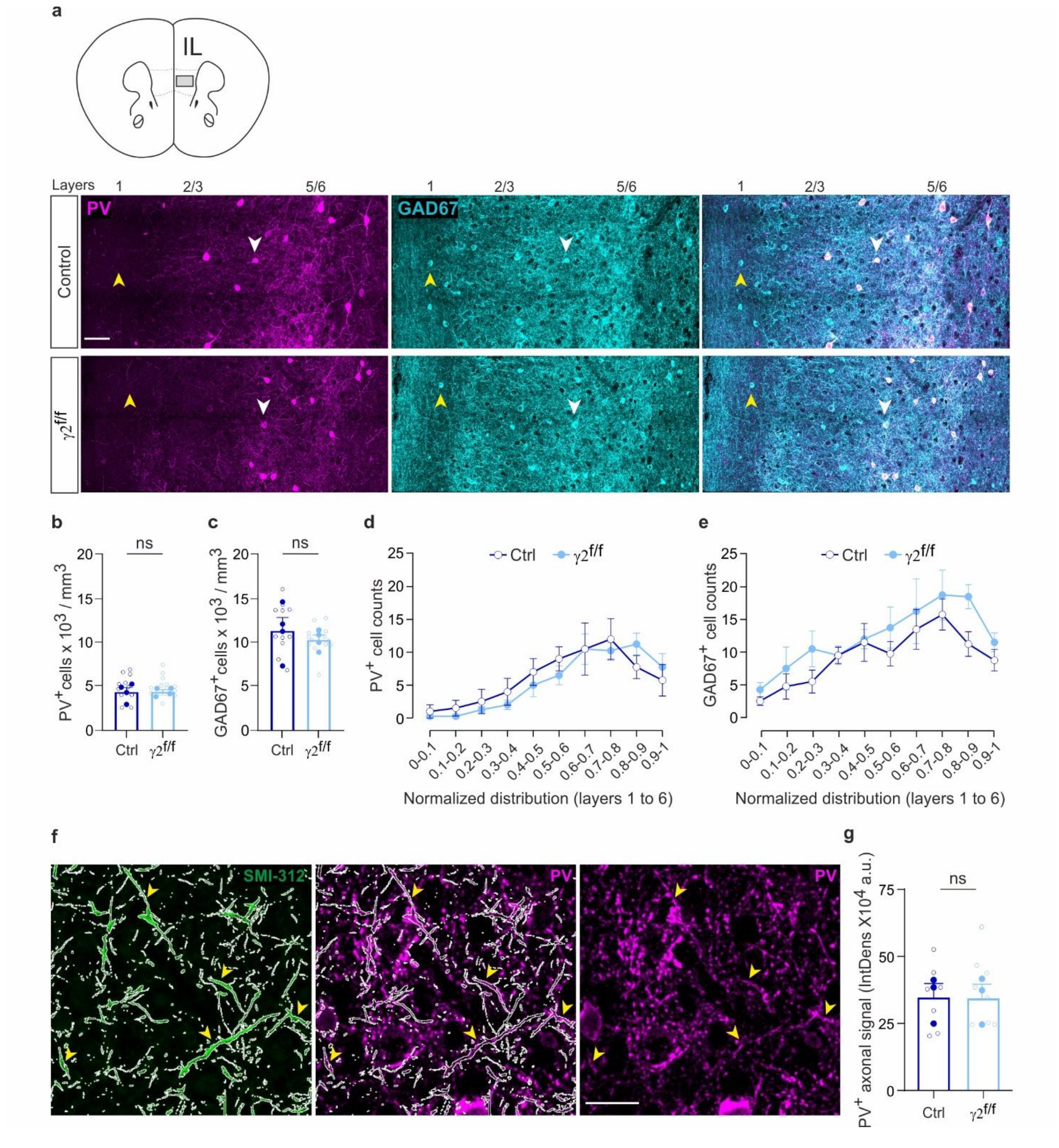

Supplementary Figure 6. No changes in the density and distribution of PV$^+$ and GAD67$^+$ interneurons of the mPFC in γ2$^{f/f}$ mice

a. Representative confocal images of PV$^+$ (magenta) and GAD67$^+$ interneurons of the IL region of the mPFC (inset) in four-month control (top) and γ2$^{f/f}$ (bottom) mice. Arrowheads indicate PV$^+$/GAD67$^+$ neurons (white) and PV$^-$/GAD67$^+$ neurons (yellow). Scale bar: 50 µm. **b-e.**

Quantification of PV^+ and $GAD67^+$ cell densities (**b, c**) and normalized distribution from layer 1 to 6 (**d, e**) in control (dark blue) and $\gamma2^{f/f}$ mice (light blue). Control mice: n= 11 technical replicates from N= 4 mice (biological replicates) and $\gamma2^{f/f}$ mice: n= 14 technical replicates from N= 4 mice (biological replicates); p= 0.719 for (**b**) and p= 0.413 for (**c**); two-tailed linear mixed models with likelihood ratio tests applied to account for repeated measures (open dots) within each animal (closed dots). For distributions, p= 0.9993 / 0.9908 / 0.9997 / 0.9950 / 0.9987 / 0.9807 / >0.9999 / >0.9999 / 0.8875 / 0.9997 for normalized bins covering layers 1-6 in (**d**) and p= >0.9999 / 0.9968 / 0.8290 / >0.9999 / >0.9999 / 0.9510 / 0.9968 / 0.9936 / 0.3770 / 0.9968 for normalized bins covering layers 1-6 in (**e**); two-way ANOVA test followed by post-hoc Sidak multiple comparisons test. Data are presented as mean±s.e.m. ns: not significant. **f.** Confocal images of SMI-312 (green) and PV (magenta) labeling, showing axonal compartments identified using a mask based on $SMI\text{-}312^+$ signal (white ROIs) in the deep layers of the IL region in a control mouse. Yellow arrowheads indicate some examples of detected PV+ axons. Scale bar: 20 µm. **g**. Dot plots of PV^+ fluorescence intensity within axonal compartment masks in control (dark blue) and $\gamma2^{f/f}$ (light blue) mice. Control mice: n= 8 technical replicates from N= 3 mice (biological replicates) and $\gamma2^{f/f}$ mice: n= 8 technical replicates from N= 3 mice (biological replicates); p=0.5 for (**g**); two-tailed linear mixed models with likelihood ratio tests applied to account for repeated measures (open dots) within each animal (closed dots). Data are presented as mean±s.e.m. ns: not significant. Source data are provided as a Source Data file.

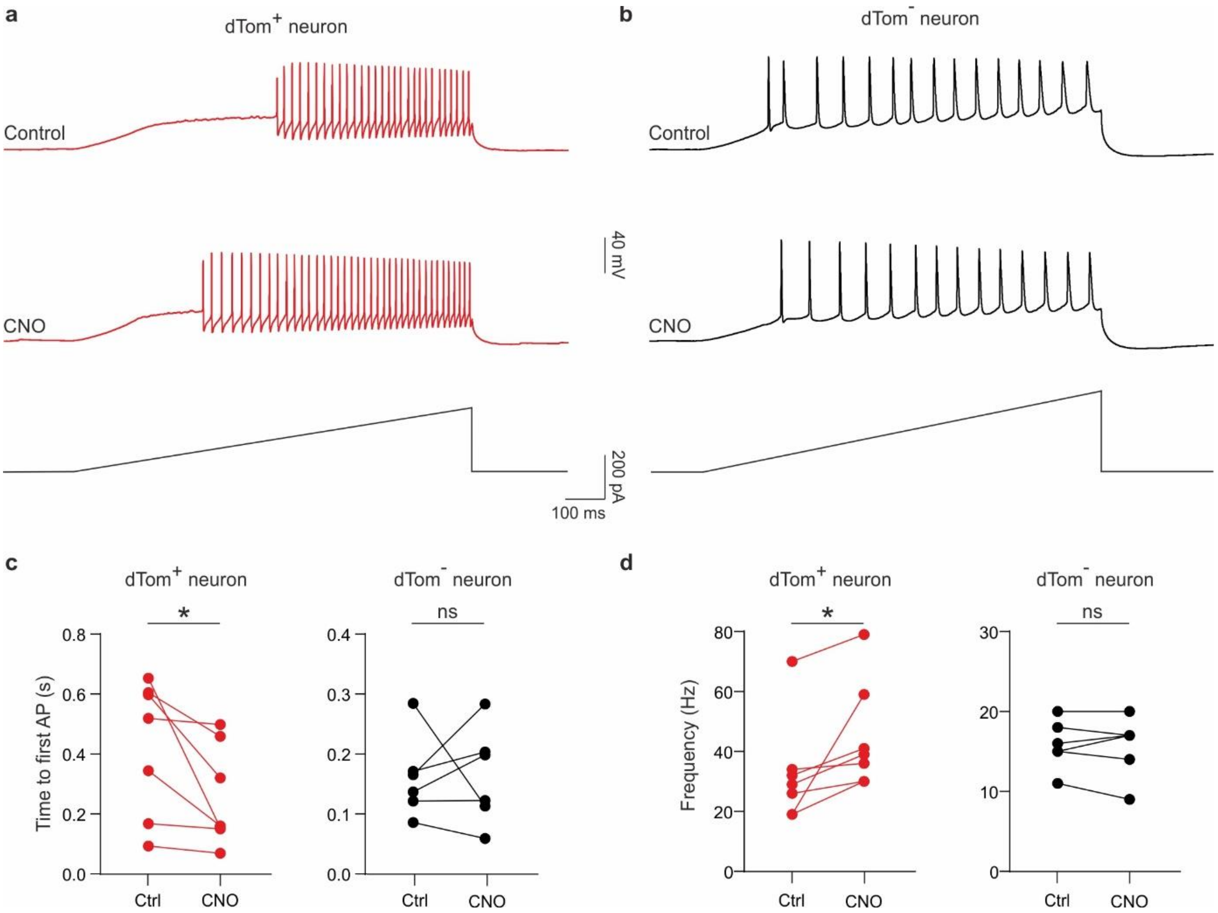

Supplementary Figure 7. Electrophysiological validation of CNO-mediated activation in hM3Dq-expressing PV interneurons.

a, b. Whole-cell patch-clamp recordings from a layer 5 dTomato^{+} PV interneurons (**a**) and a layer 2/3 dTomato^{-} pyramidal cell (**b**) in acute mPFC slices of γ2$^{f/f}$ mice injected with pAAV-S5E2-hM3D(Gq)-P2A-dTomato. Responses to depolarizing current ramps are shown before (Control, top) and after 3-min bath applications of 10 µM CNO (CNO, bottom). Note the CNO selectively reduced the latency to first action potential and increased firing frequency in the PV interneuron (red), but not in the pyramidal neuron (black). **c, d**. Dot plots of the time of first action potential (**c**) and the firing frequency (**d**) of dTomato^{+} PV interneurons (red) and neighboring dTomato^{-} pyramidal cells (black). N=3 injected γ2$^{f/f}$ mice; p= 0.0467 for dTomato^{+} PV interneurons and p= 0.9529 for dTomato^{-} pyramidal cells in (**c**), two-tailed paired Student's

t test; p= 0.0156 for $dTomato^+$ PV interneurons and p= 0.7926 for $dTomato^-$ pyramidal cells in (**d**), two-tailed Wilcoxon matched-pairs test and two-tailed paired Student's *t* test, respectively. Data are represented as mean±s.e.m. *p<0.05, ns: not significant. Ctrl: control; CNO: clozapine-N-oxide. Source data are provided as a Source Data file.

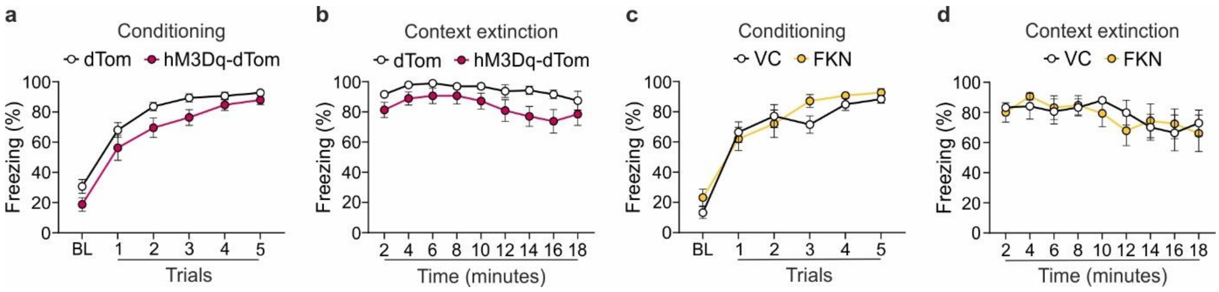

Supplementary Figure 8. Fear conditioning and context extinction sessions following chemogenetic-mediated activation of PV interneurons or in vivo FKN infusions in γ2$^{f/f}$ mice

a-d. Comparison of freezing behavior during fear conditioning and contextual extinction sessions between dTom-injected (white) and hM3Dq-dTom-injected (magenta) γ2$^{f/f}$ mice (**a, b**) and between VC-infused (white) and FKN-infused (yellow) (**c, d**). No significant differences were observed between these respective matched groups. dTom-injected γ2$^{f/f}$ mice: N=13 and hM3Dq-dTom-injected γ2$^{f/f}$ mice: N=14; VC-infused mice: N=8 and FKN-infused mice: N=8; p= 0.3668 / 0.8045 / 0.3195 / 0.2096 / 0.7111 / 0.7241 for BL and trials 1-5 in (**a**) and p= 0.4876 / 0.4341 / 0.7070 / 0.9611 / 0.6067 / 0.7291 / 0.2116 / 0.3426 / 0.9825 for 2-18 min in (**b**); p= 0.6656 / 0.9988 / 0.9988 / 0.2602 / 0.7824 / 0.8382 for BL and trials 1-5 in (**c**) and p= >0.9999 / 0.9971 / >0.9999 / >0.9999 / 0.9850 / 0.9844 / >0.9999 / >0.9999 / >0.9999 for 2-18 min in (**d**); two-way ANOVA test followed by post-hoc Sidak multiple comparisons test. Data are represented as mean±s.e.m. Source data are provided as a Source Data file.

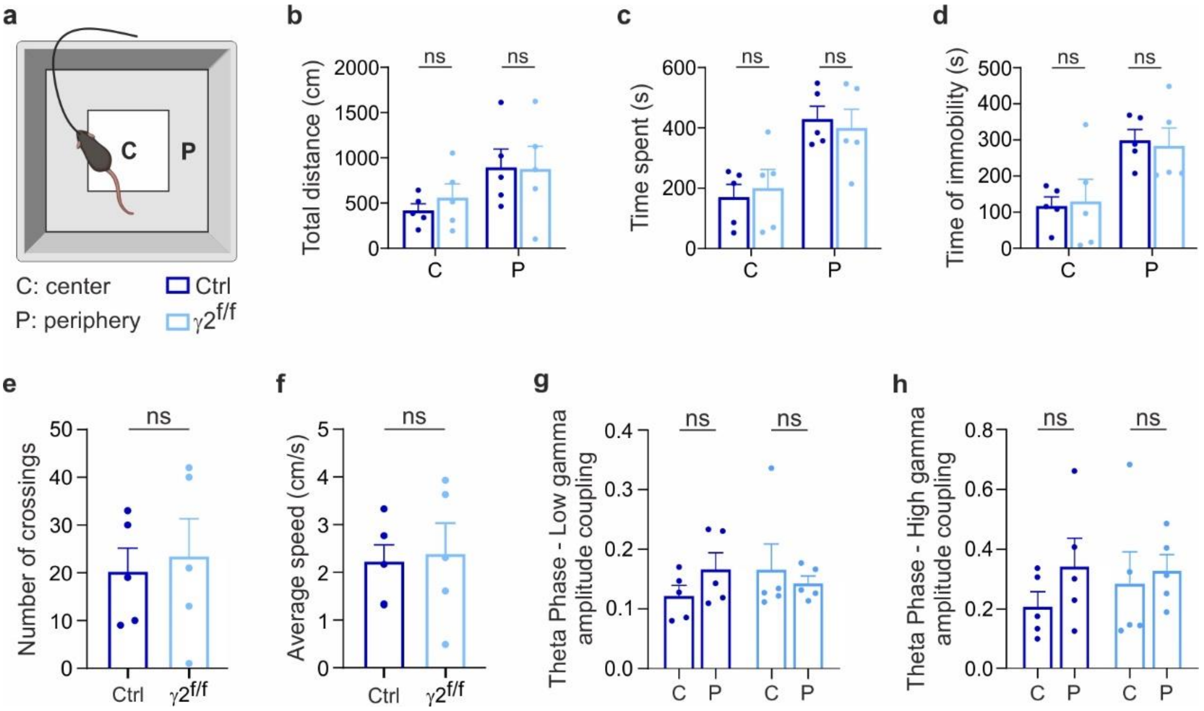

Supplementary Figure 9. Open field behavior and in vivo recordings in control and γ2$^{f/f}$ mice

a. The open-field behavior of mice was measured at the center and periphery of the arena while they were connected *via* a cable to the amplifier. **b-d.** Dot plots of the total distance traveled (**b**), time spent (**c**) and time of immobility (**d**) in the center (C) and periphery (P) of the arena for control (dark blue) and γ2$^{f/f}$ mice (light blue). N=5 control mice and N=5 γ2$^{f/f}$ mice; p= 0.4307 in center and p= 0.9553 in periphery for (**b**); p= 0.7017 in center and p= 0.7033 in periphery for (**c**); p= 0.8587 in center and p= 0.7986 in periphery for (**d**); two-tailed Student's *t* test; All data are represented as mean±s.e.m. ns: not significant. **e, f.** Dot plots of the number of crossings and average speed during the open field test in control (dark blue) and γ2$^{f/f}$ mice (light blue). N=5 control mice and N=5 γ2$^{f/f}$ mice; p= 0.7395 for (**e**); p= 0.7886 for (**f**); two-tailed Student's. All data are represented as mean±s.e.m. ns: not significant. **g-h.** Theta phase-

low-gamma amplitude (**g**) and theta phase-high-gamma amplitude (**h**) coupling did not change in the center compared to the periphery in both control (dark blue) and $\gamma2^{f/f}$ (light blue) mice. N=5 control mice and N=5 $\gamma2^{f/f}$ mice; p= 0.204 in control mice and p= 0.6165 in $\gamma2^{f/f}$ mice for (**g**); p= 0.2273 in control mice and p= 0.7238 in $\gamma2^{f/f}$ mice for (**h**); two-tailed Student's *t* test. All data are represented as mean±s.e.m; ns: not significant. Source data are provided as a Source Data file.

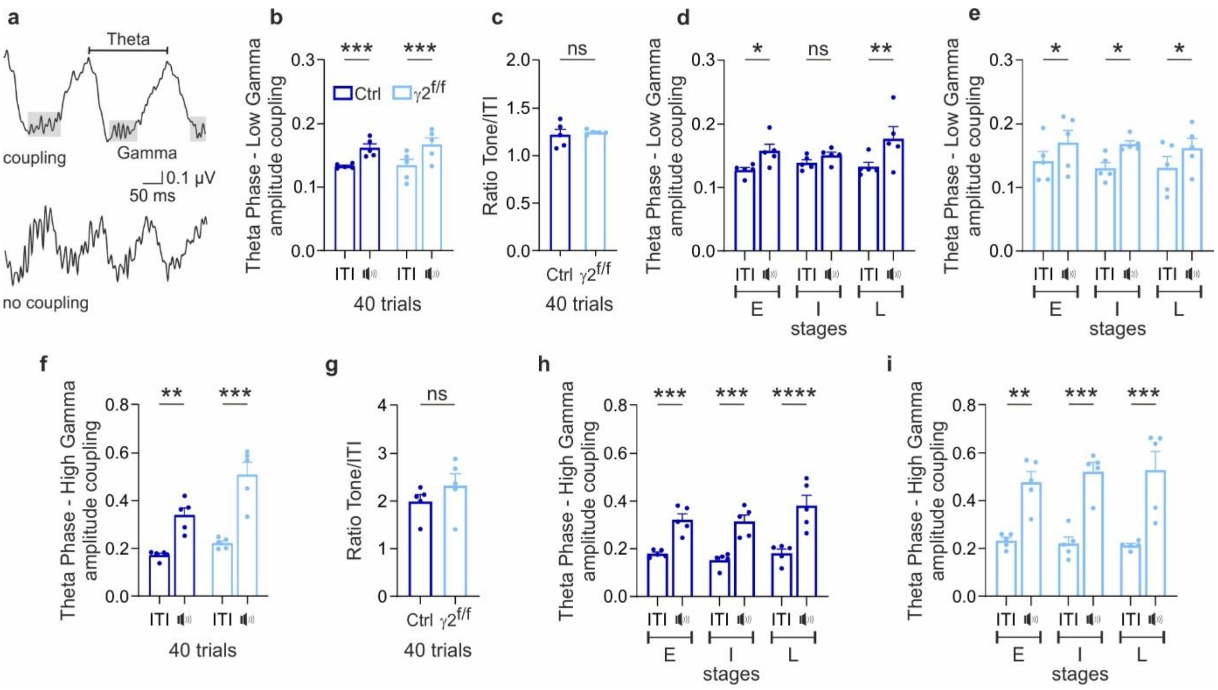

Supplementary Figure 10. No disruption of theta-high-gamma coupling during tone fear extinction

a. In vivo local field potential (LFP) recordings illustrating the coupling and uncoupling of gamma and theta oscillations. **b, f.** Theta phase-low-gamma (**b**) and theta phase-high-gamma (**f**) amplitude coupling increases during tone compared to inter-trial interval (ITI) in both control (dark blue) and $\gamma 2^{f/f}$ (light blue) mice. N=5 control mice and N=5 $\gamma 2^{f/f}$ mice. p= 0.0007 in control mice and p= 0.0003 in $\gamma 2^{f/f}$ mice for (**b**); p= 0.0041 in control mice and p= 0.0001 in $\gamma 2^{f/f}$ mice for (**f**); two-way ANOVA test followed by a Fishers Least Significant Difference (LSD) test. All data are represented as mean±s.e.m. **p<0.01, ***p<0.001. **c, g.** The ratio of theta phase-low-gamma (**c**) and theta phase-high-gamma (**g**) amplitude coupling during the tone relative to ITI is not significantly different between the two groups. N=5 control mice and N=5 $\gamma 2^{f/f}$ mice. p= 0.6585 for (**c**) and p= 0.2928 for (**g**); two-tailed Student's *t* test. All data are represented as mean±s.e.m. ns: not significant. **d-e, h-i.** Theta phase-low-gamma (**d, e**) and theta phase-high-gamma (**h, i**) amplitude coupling at early (E), intermediate (I) and late (L) stages of the extinction retrieval session in control (**d, h**) and $\gamma 2^{f/f}$ (**e, i**) mice. N=5 control mice and N=5 $\gamma 2^{f/f}$

mice. p= 0.0448 / 0.4048 / 0.0073 for early, intermediate and late stages for (**d**) and p= 0.0478 / 0.0140 / 0.0381 for early, intermediate and late stages for (**e**); p= 0.0008 / 0.0003 / <0.0001 for early, intermediate and late stages for (**h**) and p= 0.002 / 0.0004 / 0.0003 for early, intermediate and late stages for (**i**); two-way ANOVA test followed by Tukey's multiple comparison test. All data are represented as mean±s.e.m. *p<0.05, **p<0.01, ***p<0.001, ****p<0.0001, ns: not significant. Source data are provided as a Source Data file.
